# Supplementary material for: Effects of nurses’ shiftwork characteristics and aspects of private life on work-life conflict
Source: PLoS One. 2020 Dec 1;15(12):e0242379. doi: 10.1371/journal.pone.0242379 (PMC7707512; doi:10.1371/journal.pone.0242379)
Supplement: S1 File — (DOCX) [file pone.0242379.s001.docx]

아래 질문은 귀하의 개별 간호 교대근무 현황을 확인하기 위한 문항입니다.

1. 당신의 각 교대 근무의 평상 시 시간 외 근무를 하게 되는 시간을 Ⅴ로 선택해 주세요.

(a) 낮번 근무 □ 없다 □ 1시간 이내 □ 2시간 이내 □ 3시간 이내 □ 3시간 이상

(b) 초번 근무 □ 없다 □ 1시간 이내 □ 2시간 이내 □ 3시간 이내 □ 3시간

(c) 밤번 근무 □ 없다 □ 1시간 이내 □ 2시간 이내 □ 3시간 이내 □ 3시간 이상

(d) 공휴일 근무 □ 없다 □ 1시간 이내 □ 2시간 이내 □ 3시간 이내 □ 3시간 이상

(e) 그 외 근무유형(…………………………… ) (그 이외 근무 유형에 대해 자세히 써주세요)

□ 없다 □ 1시간 이내 □ 2시간 이내 □ 3시간 이내 □ 3시간 이상

2.1~2 각 항목에 해당하는 답에 Ⅴ로 표시해 주십시오.

|  | 전혀 통제할 수 없다 | 거의 통제할 수 없다 | 상당히 통제할 수 있다 | 꽤 많이 통제할 수 있다 | 완벽히 통제할 수 있다 |
| --- | --- | --- | --- | --- | --- |
| 본인이 일하게 되는 특정 교대근무 스케줄에 대해 어느 정도까지 본인이 통제할 수 있다고 느끼나요? |  |  |  |  |  |
| 본인이 교대근무를 시작하는 시간과 마치는 시간을 어느 정도까지 본인이 통제할 수 있다고 느끼나요? |  |  |  |  |  |

3. 각 항목에 해당하는 답에 Ⅴ로 표시해 주십시오.

|  | 거의 없다 | 드물게 있다 | 때때로 있다 | 자주 있다 | 거의 항상 있다 |
| --- | --- | --- | --- | --- | --- |
| (a) 짧은 시간 안에 내 근무 스케줄이 바뀌게 된 적이 있다 |  |  |  |  |  |
| (b) 동료 근무를 바뀌어 준 적이 있다 |  |  |  |  |  |
| (c) 본인이 특정 교대근무를 요청한 적이 있다 |  |  |  |  |  |

다음은 귀하의 개인 생활에 대한 질문입니다.

1. 귀하의 가족 상황은 어떠한가요?

□ 혼자 살고 있다

□ 동료, 친구, 남편 등과 함께 거주

□ 부모님과 함께 거주

□ 아이와 함께 거주

□ 남편과 아이와 함께 거주

□ 부모님, 남편, 아이와 함께 거주

2. 귀하에게 해당하는 가정에서의 요구는 무엇인가요?

(a) 돌봐야 하는 아이가 있다

□ 있다 □ 없다

(b) 집에 만성질환/장애를 가지고 있는 사람을 간병(물질적 지원 포함)하고 있다

□ 있다 □ 없다

(c) 집 밖에서 가족이나 친구를 간병(물질적 지원 포함)하고 있다

□ 있다 □ 없다

(d) 가사에 책임이 있다

□ 있다 □ 없다

3. 귀하는 가정에서의 도움이나 지지를 받고 있나요?

□ 있다 □ 없다

4. 귀하는 지난 1년간 삶의 중요한 사건을 경험하였나요?

(a) 이혼 □ 있다 □ 없다

(b) 사고 □ 있다 □ 없다

(c) 심각한 질병 □ 있다 □ 없다

(d) 중요한 사람의 죽음 □ 있다 □ 없다

5. 귀하는 **근무일**에는 집안일과 아이돌봄에 얼마나 시간을 쓰나요?

□ 0-1시간 미만

□ 1시간 이상-3시간 미만

□ 3시간 이상-5시간 미만

□ 5시간 이상-7시간 미만

□ 7시간 이상

6. 귀하는 **휴일**에는 집안일과 아이돌봄에 얼마나 시간을 쓰나요?

□ 0-1시간 미만

□ 1시간 이상-3시간 미만

□ 3시간 이상-5시간 미만

□ 5시간 이상-7시간 미만

□ 7시간 이상

7. 아래 질문은 귀하의 여가 제약에 관한 내용입니다. 해당하는 답에 Ⅴ로 표시해 주십시오.

| 문항 | 전혀그렇지 않다 | 그렇지 않다 | 보통이다 | 그렇다 | 매우 그렇다 |
| --- | --- | --- | --- | --- | --- |
| 1. 나는 여가활동을 하기에 너무 수줍음이 많다. |  |  |  |  |  |
| 2. 나는 이성과 함께 여가활동에 참가하는 것이 익숙하지 않다. |  |  |  |  |  |
| 3. 나는 주중 여가활동 참가가 어렵다. |  |  |  |  |  |
| 4. 나는 타인들과 함께 여가활동에 참가하는 것이 익숙하지 않다. |  |  |  |  |  |
| 5. 나는 공공탈의실 이용이 익숙하지 않다. |  |  |  |  |  |
| 문항 | 전혀그렇지 않다 | 그렇지 않다 | 보통이다 | 그렇다 | 매우 그렇다 |
| 6. 나는 타인이 좋은 여가활동이라고 생각하지 않는 여가활동에 참가하지 않는다. |  |  |  |  |  |
| 7. 나는 불편함을 느끼는 여가활동을 하지 않을 것이다. |  |  |  |  |  |
| 8. 여가활동에 대한 타인의 시선을 많이 의식한다. |  |  |  |  |  |
| 9. 나는 여가활동에 참가하기에 체력이 약하다. |  |  |  |  |  |
| 10. 나는 많은 기술을 요구하는 여가활동을 꺼린다. |  |  |  |  |  |
| 11. 나는 여가활동을 위한 충분한 에너지가 없다. |  |  |  |  |  |
| 12. 나는 여가활동에 함께 참가할 친구나 파트너가 없다. |  |  |  |  |  |
| 13. 내가 여가활동에 같이 참여하고 싶은 사람은 너무 멀리 떨어져 있다. |  |  |  |  |  |
| 14. 내가 여가활동에 같이 참가하고 싶은 사람들은 여가활동을 위한 충분한 시간이 없다. |  |  |  |  |  |
| 15. 내가 여가활동에 같이 참가하고 싶은 사람들은 충분한 돈이 없다. |  |  |  |  |  |
| 16. 내가 여가활동에 같이 참가하고 싶은 사람들은 일(가사)이 너무 많다. |  |  |  |  |  |
| 17. 내가 여가활동에 같이 참가하고 싶은 사람들은 여가활동에 대한 충분한 지식이나 기술이 없다. |  |  |  |  |  |
| 18. 내가 여가활동에 같이 참가하고 싶은 사람들은 나와 시간대가 맞지 않는다. |  |  |  |  |  |
| 19. 나는 사람들이 붐비는 여가활동을 미루거나 생략한다. |  |  |  |  |  |

| 문항 | 전혀그렇지 않다 | 그렇지 않다 | 보통이다 | 그렇다 | 매우 그렇다 |
| --- | --- | --- | --- | --- | --- |

| 20. 나는 다른 일이 있을 때 여가활동을 미루거나 생략한다. |  |  |  |  |  |
| --- | --- | --- | --- | --- | --- |
| 21. 나는 여가활동 참가에 필요한 복장과 장비가 없다. |  |  |  |  |  |
| 22. 나는 좋지 않은 시설에서의 여가활동을 꺼린다. |  |  |  |  |  |
| 23. 여가활동에 관한 정보가 너무 부족하다. |  |  |  |  |  |
| 24. 여가활동을 하기에 주변 환경이 적절하지 않다. |  |  |  |  |  |
| 25. 여가활동비용이 너무 많이 든다. |  |  |  |  |  |
| 26. 나는 여가활동에 참가할 충분한 시간이 없다. |  |  |  |  |  |
| 27. 나는 일(가사)로 너무 바쁘다. |  |  |  |  |  |
| 28. 나의 신체적 조건이 여가활동을 방해한다. |  |  |  |  |  |

아래 질문은 귀하의 일 ∙ 생활 갈등 관한 내용입니다. 해당하는 답에 Ⅴ로 표시해 주십시오.

| 문항 | 전혀그렇지 않다 | 그렇지 않다 | 보통이다 | 그렇다 | 매우 그렇다 |
| --- | --- | --- | --- | --- | --- |
| 1. 나는 개인생활(가족)에 더 많은 시간을 보내고 싶지만, 직장일로 인해서 그러지 못한다. |  |  |  |  |  |
| 2. 직장일에 쏟아야 하는 시간 때문에 개인(가정)에서의 책임을 이행하거나 개인 생활(가정행사)을 이행하는 데 지장이 있다. |  |  |  |  |  |
| 3. 직장에서 책임을 완수하는데 보내는 시간 때문에 개인 생활(가족과 함께 하는 활동)을 놓친다. |  |  |  |  |  |
| 문항 | 전혀그렇지 않다 | 그렇지 않다 | 보통이다 | 그렇다 | 매우 그렇다 |
| 4. 개인(가정)의 책임을 이행하는데 보내는 시간 때문에 나의 업무는 종종 방해를 받는다. |  |  |  |  |  |
| 5. 개인적인 생활(가족과 보내는 시간) 때문에 내 경력에 도움이 될 만한 직장 내 활동을 할 시간을 내지 못하는 경우가 종종 있다. |  |  |  |  |  |
| 6. 개인(가족) 책임을 이행하는데 할애해야만 하는 시간이 있기 떄문에 직장 내 활동을 놓치게 된다. |  |  |  |  |  |

다음은 귀하의 근무지 특성과 일반적 사항에 관한 질문입니다.

1. 귀하는 현재 어느 부서에서 근무하고 계십니까?

□ 내과계 일반병동 □ 외과계 일반병동 □ 중환자실 □ 응급실 □ 간호간병통합서비스병동

2. 귀하의 성별은? □ 여자 □ 남자

3. 귀하의 혼인 상태는? □ 미혼 □ 기혼

4. 귀하의 연령은? 만 ( ) 세

5. 귀하의 최종 학력은? □ 전문학사(전문대 학위) □ 학사학위 □ 석사학위 □ 박사학위

6. 귀하께서는 간호사로 얼마 동안 근무하셨습니까? ( ) 년 ( ) 개월

7. 귀하께서는 현재 병원에 얼마 동안 근무하셨습니까? ( ) 년 ( ) 개월

8. 귀하의 월평균 소득(세금공제 후 소득)은? ( )만원
