# Supplementary material for: Effects of nurses’ shiftwork characteristics and aspects of private life on work-life conflict
Source: PLoS One. 2020 Dec 1;15(12):e0242379. doi: 10.1371/journal.pone.0242379 (PMC7707512; doi:10.1371/journal.pone.0242379)
Supplement: S2 File — (DOCX) [file pone.0242379.s002.docx]

Please answer the following questions of shiftwork characteristics as accurately as possible.

1. On average, how many hours overtime do you work each week? (Circle one)

(a) Morning shift

□ none □ within 1 hour □ 1 to within 2 hours □ 2 to within 3 hours □ above 3 hours

(b) Evening shift

□ none □ within 1 hour □ 1 to within 2 hours □ 2 to within 3 hours □ above 3 hours

(c) Night shift

□ none □ within 1 hour □ 1 to within 2 hours □ 2 to within 3 hours □ above 3 hours

(d) Holiday shift

□ none □ within 1 hour □ 1 to within 2 hours □ 2 to within 3 hours □ above 3 hours

(e) Other (…………………………… ) (please specify)

□ none □ within 1 hour □ 1 to within 2 hours □ 2 to within 3 hours □ above 3 hours

2.1 To what extent do you feel you have control over the specific shifts that you work?

| None | Not very much | A fair amount | Quite a lot | Complete |
| --- | --- | --- | --- | --- |
| 1 | 2 | 3 | 4 | 5 |

2.2. To what extent do you have control of the specific start and finish times of the shifts you work?

| None | Not very much | A fair amount | Quite a lot | Complete |
| --- | --- | --- | --- | --- |
| 1 | 2 | 3 | 4 | 5 |

3. For each of the following, please indicate how often you: (please circle one number for each)

|  | Almost never | Rarely | Sometimes | Frequently | Almost always |
| --- | --- | --- | --- | --- | --- |
| (a) Are required to change your roster at short notice |  |  |  |  |  |
| (b) Swop shifts with collegues |  |  |  |  |  |
| (c) Make a request to work specific shifts |  |  |  |  |  |

Please answer the following questions of aspects of private life as accurately as possible.

1. Family status

□ Living alone

□ Living with co-workers and friends, etc.

□ Living with parents

□ Living with children

□ Living with partner (including husband or wife) and children

□ Living with parents, partner and children

2. What is your demands at home?

(a) Dependent children

□ Yes □ No

(b) Care chronically ill person at home

□ Yes □ No

(c) Responsible care family/friend outside home

□ Yes □ No

(d) Responsibility housekeeping

□ Yes □ No

3. Do you have a domestic help?

□ Yes □ No

4. Do you have life events during past year?

(a) Divorce □ Yes □ No

(b) Accident □ Yes □ No

(c) Severe illness □ Yes □ No

(d) Death of an important person □ Yes □ No

5. How many times do you spent on housework and child rearing on workdays?

□ 0 to within 1 hour

□ 1 to within 3 hours

□ 3 to within 5 hours

□ 5 to within 7 hours

□ Above 7 hours

6 How many times do you spent on housework and child rearing on holidays?

□ 0 to within 1 hour

□ 1 to within 3 hours

□ 3 to within 5 hours

□ 5 to within 7 hours

□ Above 7 hours

Please answer the following questions of leisure constraints as accurately as possible.

| Items | Almost never | Rarely | Sometimes | Frequently | Almost always |
| --- | --- | --- | --- | --- | --- |
| 1. I am too shy to start a leisure activity. |  |  |  |  |  |
| 2. I am not used to participating in leisure activities with opposite sex. |  |  |  |  |  |
| 3. It is difficult for me to participate in the leisure activities during weekdays. |  |  |  |  |  |
| 4. I am not used to participating in leisure activities with others. |  |  |  |  |  |
| 5. I'm not used to using public dressing rooms. |  |  |  |  |  |
| 6. I am unlikely to do a leisure activity that my friends thought was not alright. |  |  |  |  |  |
| 7. I am unlikely to do a leisure activity that makes me feel uncomfortable. |  |  |  |  |  |
| 8. I am conscious of other people's views on leisure activities |  |  |  |  |  |
| 9. I am too weak to start a leisure activity. |  |  |  |  |  |
| 10. I am unlikely to do a leisure activity that does required a lot of skills. |  |  |  |  |  |
| 11. I do not have enough energy to start a leisure activity. |  |  |  |  |  |
| 12. I do not have friends or partners to start a leisure activity with me. |  |  |  |  |  |
| 13. The people I know live too far away to start a leisure activity with me. |  |  |  |  |  |
| 14. The people I know usually don’t have time to start a leisure activity with me. |  |  |  |  |  |
| 15. The people I know usually have enough money to begin a leisure activity with me. |  |  |  |  |  |
| 16. The people I know usually have too many work (family) obligations to start a lesiure activity with me. |  |  |  |  |  |
| 17. The people I know usually don’t have enough knowledges and skills to start a leisure activity with me. |  |  |  |  |  |
| 18. The people I know usually don’t fit the time to start a leisure activity with me. |  |  |  |  |  |
| 19. I am more likely to a leisure activity if the facilities I need to do the activity are not crowded. |  |  |  |  |  |
| 20. I am unlikely to do a leisure activity if I have other commitments. |  |  |  |  |  |
| 21. I do not have enough goods to start a leisure activity. |  |  |  |  |  |
| 22. I am unlikely to do a leisure activity in poor facilities. |  |  |  |  |  |
| 23. I do not have enough informations of a leisure activity. |  |  |  |  |  |
| 24. I am unlikely to do a leisure activity if the environments are not proper. |  |  |  |  |  |
| 25. I am unlikely to do a leisure activity if I don’t’ have money. |  |  |  |  |  |
| 26. I am unlikely to a leisure activity if I don’t have time. |  |  |  |  |  |
| 27. I am too busy with work (housework). |  |  |  |  |  |
| 28. My physical condition interferes with leisure activities. |  |  |  |  |  |

Please answer the following questions of work-life conflict as accurately as possible.

| Items | Almost never | Rarely | Sometimes | Frequently | Almost always |
| --- | --- | --- | --- | --- | --- |
| 1. My work keeps me from my family activities more than I would like. |  |  |  |  |  |
| 2. The time I must devote to my job keeps me from participating equally in household responsibilities and activities. |  |  |  |  |  |
| 3. I have to miss family activities due to the amount of time I must spend on work responsibilities. |  |  |  |  |  |
| 4. The time I spend on family responsibilities often interfere with my work responsibilities. |  |  |  |  |  |
| 5. The time I spend with my family often causes me not to spend time in activities at work that could be helpful to my career. |  |  |  |  |  |
| 6. I have to miss work activities due to the amount of time I must spend on family responsibilities. |  |  |  |  |  |

Please answer the following questions of work unit and general characteristics

1. What is your department?

□ Medical ward □ Surgical ward □ Intensive care unit

□ Emergency room □ Nursing care integrated services ward

2. Sex? □ female □ male

3. Marital status? □ Single □ Married

4. Age? ( ) years

5. Education? □ Associate degree □ Bachelor’s degree □ Master’s degree or higher

6. Work experiences as a nurse? ( ) years

7. Current work experience? ( ) years

8. Average income per a month? ( ) Million Won
