# Supplementary material for: Effects of nurses’ shiftwork characteristics and aspects of private life on work-life conflict
Source: PLoS One. 2020 Dec 1;15(12):e0242379. doi: 10.1371/journal.pone.0242379 (PMC7707512; doi:10.1371/journal.pone.0242379)
Supplement: Attachment — Submitted filename: PONE-D-20-18345_reviewered.pdf [file pone.0242379.s003.pdf]

PLOS ONE

Effects of nurses' shiftwork characteristics and aspects of private life on work-life conflict

--Manuscript Draft--

**Manuscript Number:**	PONE-D-20-18345
**Article Type:**	Research Article
**Full Title:**	Effects of nurses' shiftwork characteristics and aspects of private life on work-life conflict
**Short Title:**	Effects of nurses' shiftwork characteristics on work-life conflict
**Corresponding Author:**	Hye-Kyung Oh<br>Seoul National University<br>Seoul, KOREA, REPUBLIC OF
**Keywords:**	nurses; shiftwork characteristics; private life; work-life conflict
**Abstract:**	Background<br><br>As nurses must work highly irregular hours, the characteristics of shiftwork and aspects of their private lives are important factors that may contribute significantly to work-life conflict.<br><br>Purpose: This study examined the effects of nurses' shiftwork characteristics and aspects of their private lives on work-life conflict.<br><br>Methods<br><br>The sample included 271 registered nurses working three-shifts rotations in five types of units at four hospitals in South Korea. We administered structured questionnaires regarding shiftwork characteristics, private life, and work-life conflict. Data were analyzed using multiple linear regression analysis.<br><br>Results<br><br>The significant factors relating to work-life conflict included control over shift start and finish times (β=-0.16, p =.019) and frequency of swapping shifts with colleagues (β=0.15, p =.025) among shiftwork characteristics, and leisure constraints (β=0.39, p =<.001) among aspects of private life.<br><br>Conclusion<br><br>Plan and policies for improving nursing environments should focus on improving nurses' control over shiftwork and decreasing leisure constraints.
**Order of Authors:**	Hye-Kyung Oh
	Sung-Hyun Cho
**Additional Information:**	
**Question**	**Response**
**Financial Disclosure**<br><br>Enter a financial disclosure statement that describes the sources of funding for the work included in this submission. Review the submission guidelines for detailed requirements. View published research articles from *PLOS ONE* for specific examples.	The authors received no specific funding for this work

This statement is required for submission and **will appear in the published article** if the submission is accepted. Please make sure it is accurate.<br><br>**Unfunded studies**<br>Enter: *The author(s) received no specific funding for this work.*<br><br>**Funded studies**<br>Enter a statement with the following details:<br>• Initials of the authors who received each award<br>• Grant numbers awarded to each author<br>• The full name of each funder<br>• URL of each funder website<br>• Did the sponsors or funders play any role in the study design, data collection and analysis, decision to publish, or preparation of the manuscript?<br>• **NO** - Include this sentence at the end of your statement: *The funders had no role in study design, data collection and analysis, decision to publish, or preparation of the manuscript.*<br>• **YES** - Specify the role(s) played.<br><br>* typeset	
**Competing Interests**<br><br>Use the instructions below to enter a competing interest statement for this submission. On behalf of all authors, disclose any competing interests that could be perceived to bias this work—acknowledging all financial support and any other relevant financial or non-financial competing interests.<br><br>This statement **will appear in the published article** if the submission is accepted. Please make sure it is accurate. View published research articles from *PLOS ONE* for specific examples.	No authors have competing interests

Powered by Editorial Manager® and ProduXion Manager® from Aries Systems Corporation

**NO authors have competing interests**<br><br>Enter: *The authors have declared that no competing interests exist.*<br><br>**Authors with competing interests**<br><br>Enter competing interest details beginning with this statement:<br><br>*I have read the journal's policy and the authors of this manuscript have the following competing interests: [insert competing interests here]*<br><br>* typeset	
**Ethics Statement**<br><br>Enter an ethics statement for this submission. This statement is required if the study involved:<br><br>• Human participants<br>• Human specimens or tissue<br>• Vertebrate animals or cephalopods<br>• Vertebrate embryos or tissues<br>• Field research<br><br>Write "N/A" if the submission does not require an ethics statement.<br><br>General guidance is provided below. Consult the submission guidelines for detailed instructions. **Make sure that all information entered here is included in the Methods section of the manuscript.**	This sutdy was approved by the bioethics committee of Seoul Natioanl University (IRB NO. 1909/001-001)

Powered by Editorial Manager® and ProduXion Manager® from Aries Systems Corporation

**Format for specific study types**<br><br>**Human Subject Research (involving human participants and/or tissue)**<br>• Give the name of the institutional review board or ethics committee that approved the study<br>• Include the approval number and/or a statement indicating approval of this research<br>• Indicate the form of consent obtained (written/oral) or the reason that consent was not obtained (e.g. the data were analyzed anonymously)<br><br>**Animal Research (involving vertebrate animals, embryos or tissues)**<br>• Provide the name of the Institutional Animal Care and Use Committee (IACUC) or other relevant ethics board that reviewed the study protocol, and indicate whether they approved this research or granted a formal waiver of ethical approval<br>• Include an approval number if one was obtained<br>• If the study involved *non-human primates*, add *additional details* about animal welfare and steps taken to ameliorate suffering<br>• If anesthesia, euthanasia, or any kind of animal sacrifice is part of the study, include briefly which substances and/or methods were applied<br><br>**Field Research**<br><br>Include the following details if this study involves the collection of plant, animal, or other materials from a natural setting:<br>• Field permit number<br>• Name of the institution or relevant body that granted permission	
**Data Availability**<br><br>Authors are required to make all data underlying the findings described fully available, without restriction, and from the time of publication. PLOS allows rare exceptions to address legal and ethical concerns. See the PLOS Data Policy and FAQ for detailed information.	Yes - all data are fully available without restriction

A Data Availability Statement describing where the data can be found is required at submission. Your answers to this question constitute the Data Availability Statement and **will be published in the article**, if accepted.<br><br>**Important:** Stating 'data available on request from the author' is not sufficient. If your data are only available upon request, select 'No' for the first question and explain your exceptional situation in the text box.<br><br>Do the authors confirm that all data underlying the findings described in their manuscript are fully available without restriction?	
**Describe where the data may be found in full sentences. If you are copying our sample text, replace any instances of XXX with the appropriate details.**<br><br>• If the data are **held or will be held in a public repository**, include URLs, accession numbers or DOIs. If this information will only be available after acceptance, indicate this by ticking the box below. For example: *All XXX files are available from the XXX database (accession number(s) XXX, XXX.).*<br>• If the data are all contained **within the manuscript and/or Supporting Information files**, enter the following: *All relevant data are within the manuscript and its Supporting Information files.*<br>• If neither of these applies but you are able to provide **details of access elsewhere**, with or without limitations, please do so. For example:<br><br>*Data cannot be shared publicly because of [XXX]. Data are available from the XXX Institutional Data Access / Ethics Committee (contact via XXX) for researchers who meet the criteria for access to confidential data.*<br><br>*The data underlying the results presented in the study are available from (include the name of the third party*	https://www.protocols.io/view/untitled-protocol-bhihj4b6

and contact information or URL).<br>• This text is appropriate if the data are owned by a third party and authors do not have permission to share the data.<br><br>* typeset	
Additional data availability information:	Tick here if the URLs/accession numbers/DOIs will be available only after acceptance of the manuscript for publication so that we can ensure their inclusion before publication.

Powered by Editorial Manager® and ProduXion Manager® from Aries Systems Corporation

Manuscript

Click here to access/download;Manuscript;Manuscript (17 June).docx

Effects of nurses' shiftwork characteristics and aspects of private life on work-life conflict

Hye-Kyung Oh[1] *, Sung-Hyun Cho[1]

[1]College of Nursing, Seoul National University, Seoul, South Korea

* Corresponding author: Hye-Kyung Oh

E-mail: katie5@snu.ac.kr

Abstract

Background

As nurses must work highly irregular hours, the characteristics of shiftwork and aspects of their private lives are important factors that may contribute significantly to work-life conflict. Purpose: This study examined the effects of nurses' shiftwork characteristics and aspects of their private lives on work-life conflict.

Methods

The sample included 271 registered nurses working three-shifts rotations in five types of units at four hospitals in South Korea. We administered structured questionnaires regarding shiftwork characteristics, private life, and work-life conflict. Data were analyzed using multiple linear regression analysis.

Results

The significant factors relating to work-life conflict included control over shift start and finish times (β=-0.16, *p*=.019) and frequency of swapping shifts with colleagues (β=0.15, *p*=.025) among shiftwork characteristics, and leisure constraints (β=0.39, *p*=<.001) among aspects of private life.

Conclusion

Plan and policies for improving nursing environments should focus on improving nurses' control over shiftwork and decreasing leisure constraints.

Introduction

Background

Nurses are a frontline occupational group who work with patients throughout the daily 24-hour period. Having to work irregular hours frequently, however, could severely impact work-life balance [1,2]. Adjusting work schedules can be a solution to improve work-life balance [3]. Currently, there exists a serious shortage of nurses in small and medium-sized hospitals in South Korea [4]. This does not mean that there is necessarily an overall shortage in the total number of nurses. However, there are mismatches in the supply and demand of nurses. To resolve this situation, the Korean Ministry of Education proposed an alternative plan: to transfer university students to nursing colleges to increase the number of nursing students from 10% to 30%. Under this scheme, students who wanted to pursue nursing courses at a nursing college were transferred [5]. However, the critical problem is that a large number of registered nurses are currently out of employment. Therefore, the fundamental causes leading to the mismatch in supply and demand of nurses have to be identified and rectified.

The primary reason for the large proportion of idle nurses is irregular working hours unsuitable for pregnancy and childcare, such as three-shift schedules. This is also impacted by social environments where work-life balance is difficult [6]. Shiftwork is inevitable for providing continuous, high-quality nursing care. However, there are limitations to adjusting shiftwork schedules among limited numbers of nurses in a way that reflects a variety of personal preferences. As optimal shiftwork schedules that satisfy everyone have not been developed, it is important to obtain basic data to identify the facts associated with shiftwork characteristics and to identify the relevant variables that should be incorporated into policy suggestions that can improve the current situation.

In the USA, the National Institute for Occupational Safety and Health (NIOSH) had conducted three waves of studies from 1993 to 2006 on various issues related to nurses, including shiftwork characteristics [7]. As part of the studies, shiftwork characteristics were explored using items of the Standard Shiftwork Index (SSI) in the form of a questionnaire used internationally. Using the data from these studies, researchers looked into conditions of working hours, overtime, etc. and also analyzed non-work activities such as housework, childcare chores, and leisure time [7]. As such a study has not been conducted in Korea, we deemed it necessary to analyze the shiftwork characteristics and aspects of the private life of nurses in the country.

In previous studies, nurses' working hours, overtime [8,9], and flexible working hours [8] were found to be related to work-life conflict. Irregular shiftwork schedules and long working hours were found to be the most important factors in increasing work-life conflict [9].

Childcare and time spend on domestic chores were activities of private life that were found to interfere with work. While there have been many studies in various countries incorporating objective variables such as shiftwork schedule analysis and work pattern investigations, most of the studies in Korea have been based on nurse recognition surveys. Hence, further research is needed to examine the effects of nurses' shiftwork characteristics and aspects of private life on work-life conflict.

Materials and methods

Setting and Sample

The study sample included 271 shift nurses working in five units (medical, surgical, intensive care, emergency, and integrated nursing care units) at four hospitals. The subjects were selected randomly based on their willingness to participate in the study voluntarily. 280

subjects were initially recruited, with consideration of the expected dropout rate. After the completed questionnaires were received, a preprocessing check was conducted to remove incomplete or invalid data.

Variables and instruments

Shiftwork characteristics were measured using 11 variables. Some of these variables were measured using the Standard Shiftwork Index developed by Barton. et al. [10]. Other variables that were measured using items relating to shiftwork characteristics are provided in Table 1. Since the Standard shiftwork index is based on self-reporting, concerns have been raised about inaccuracies and response bias relating to participants' memory. To compensate for this, in the current study, unit shiftwork schedules were directly verified by the researcher. Variables that the researcher directly collected data on the included number of over 40-hour working weeks in a month, unsocial working hours per week, number of maximum consecutive shifts worked during the previous three months, number of unhealthy shift patterns in a month, and nurse staffing adequacy.

To verify the average number of unhealthy shift patterns in a month, different types of unhealthy working patterns were enumerated by the researcher with reference to health management for shift workers of Korean Ministry of Employment and Labor [11], and the opinion on recommendations for working guidelines for healthy shift workers of Korean Confederation of Trade Unions [12]. After these items were developed, three professors of nursing management and three nurses with over ten years of clinical experience conducted a content validity survey (CVI), to arrive at eight unhealthy shift patterns comprising items with validity scores of 0.8 or higher. These items were: double shifts, working three-shift rotations for more than five consecutive days, working more than seven nights in a month, working more than three consecutive night shifts, returning to work without a 48-hour rest

period after night shifts, less than 24 hours rest period between shifts, less than two nurses working on night shifts, and backward rotating working system.

The formula for calculating nurse staffing adequacy was the sum of the total number of nurses assigned during a 24-hour working day divided by the total number of required nurses for 24 hours of the same day. The number of nurses required for each unit was calculated according to the Korean graded fee of nursing management, the criteria for deploying staffing in the integrated nursing care unit, and the number of nurses in emergency rooms for each emergency medical institution. The calculation of the number of nurses required by each unit incorporated the three-shift rotation, 40-hour work per week, vacations, and days-off. Therefore, 1.6 was divided to obtain the total number of nurses required per day [13,14]. An example of a nurse staffing adequacy calculation is as follows (Figure 1). As the hospital was a general hospital and had a second graded fee of nursing management (general ward), the ratio of the number of nurses and beds should be between 1:2.5 and less than 3.0. The number of beds in this ward was 54 beds. Therefore, 19 nurses are required as a minimum to satisfy the second-grade fee of nursing management. Among the 19 nurses, the required number of nurses per day was 11.875 (about 12) from the 1.6 division and by considering the three-shift rotation, 40-hour working per week, and holidays. In the case of nurse 1, they worked 11 days, and multiplying the required number of nurses by 12 for each workday would be result in a total of 132 nurses. The total number of nurses assigned on the workdays was 78, and therefore only 59% of the required number of nurses were at work.

Table 1. Research variables for shiftwork characteristics, aspects of private life, and work-life conflict

Categories	Variables	Explanation	Instrument
Work hours	Average number of over 40-hour working weeks in a month	Average number of nurses who are scheduled to work for over 40 hours per week	SSI
	Unsocial working hours per week	Unsocial work hours per week (night work: 10 pm to 5am, evening work: 6 pm to 10 pm, and Saturday /Sunday work)	European unsocial hours
	Overtime	Amount of overtime work per shift	SSI
Shift patterns	Number of maximum consecutive shifts	Number of maximum consecutive shifts between days off over 3 months	SSI
	Average number of unhealthy shift patterns in a month	Average number of nurses who are assigned unhealthy shift patterns per month	
Nurse staffing adequacy	Nurse staffing adequacy (%)	Ratio of the number of nurses required in the ward to the number of nurses who actually work there.	
Shift stability	Control over specific shifts	5-point scale	SSI
	Control of specific start & finish times		
Shift instability	Required to change shifts at short notice		
	Swapping shifts with colleagues		
	Requests to work specific shifts		
Characteristics of private life	Family status	Current living arrangements of the subject	Previous studies
	Demands at home	Dependent children, caring for someone with a chronic disease/handicap, caring for family/friend, housekeeping responsibility, presence of life events	
	Time spent on housework or child rearing	Time spent on housework or child rearing on work /holidays	
	Support at home	Whether domestic help is available or not	
Leisure constraints	Leisure constraints	Intrapersonal/interpersonal/structural constraints	Leisure constraints
Work-life conflict	Work-life conflict	(Time based) Work interference with life, Life interference with work	Work-life conflict

Aspects of private life were measured using item developed by Jansen, Kant, Kristensen, and Nijhuis [15], Takeuchi and Yamazaki [16], and Raymore, Godbey, Crawford, and Von Eye [17], which were modified by Hubbard and Mannell [18] and Hwang and Seo [19]. This measure consisted of five variables: family status, demands at home, time spent on housework and child-rearing, support at home, and leisure constraints. Family status, demands at home, and support at home were measured as dichotomous (yes or no). Time spent on housework and child-rearing (workday and holiday) was measured on a 5-point scale (0 to more than 7 hours for workdays, 0 to more than 12 hours for holidays). Leisure constraints consisted of intrapersonal, interpersonal, and structural constraints, and they were measured on a 5-point scale ("not at all" to "very much").

The variable of work-life conflict was measured using a scale developed by Carlson, Kacmar, and Williams [20]. It consisted of two elements (work interference with life, life interference with work). Each question used a 5-point scale ("not at all" to "very much") to measure response.

Data collection

Nurse manager' and nurse' surveys were conducted from August to September 2019. In the nurse manager survey, managers reported their unit type, the number of beds, and the average number of patients at midnight. Objective variables of the shiftwork characteristics of nurses were collected from the units' shiftwork schedules for the previous three months. This information was used to compute the average patients/beds-to-RN ratio and other shiftwork characteristics. Nurses participated in the nurse survey via written questionnaires. We ensured that nurse managers were unable to identify whether individual nurses had participated or not. Data collection was undertaken after obtaining approval from the institutional review board.

To ensure anonymity, nurses' names were deleted from the unit shiftwork schedules by nurse' managers and replaced with serial numbers matched between the shiftwork schedules and questionnaires.

Data analysis

All collected data were analyzed using SPSS Statistics 22.0 (IBM Corp., Armonk, MY, USA). We described nurses' demographic characteristics, shiftwork characteristics, aspects of private life, and work-life conflict using real numbers, percentages, means, and standard deviations. The factors influencing work-life conflicts were analyzed using multiple linear regression analyses.

Results

General characteristics

Table 2 shows the general characteristics of 217 participating nurses. Most were female (88.2%) and more than 70% were single or unmarried. In terms of age, the largest proportion belonged to the 20-29 age group (57.9%). Thirty percent of the nurses worked in the integrated nursing care unit, while 29.5%, 17.7%, 14.8%, and 7.4% worked in the medical, emergency, intensive care, and surgical units, respectively. Approximately two-thirds held a baccalaureate or higher degree, and the respondents had worked as an RN for 6.6 years on average. In terms of monthly income, the majority of 76% earned between 2million and 3 million won.

Table 2. General characteristics of participants (n=271)

Category		Frequency	Percent
Sex	Male	32	11.8
	Female	239	88.2
Marital status	Single	191	70.5
	Married	78	28.8
	Divorced	2	0.7
Age (Mean 29.61)	20-29	157	57.9
	30-39	88	32.5
	≥ 40	26	9.6
Unit type	Medical ward	80	29.5
	Surgical ward	20	7.4
	Intensive care unit	40	14.8
	Emergency room	48	17.7
	Nursing care integrated services ward	83	30.6
Education	Associate degree	94	34.8
	Bachelor's degree	172	63.7
	Master's degree or higher	4	1.5
Work experience (Mean 6.64)	< 5 years	137	50.6
	5≤~<10 years	68	25.1
	10≤~<15 years	35	12.9
	15≤~<20 years	22	8.1
	≥ 20 years	9	3.3
Current work experience (Mean 4.76)	< 5 years	174	64.2
	5≤~<10 years	65	24.0
	10≤~<15 years	18	6.6
	15≤~<20 years	7	2.6
	≥ 20 years	6	2.2
Average income (Mean 2.47 Million Won)	Less than 2 Million Won	18	6.6
	Over 2~Less than 2.5 Million Won	107	39.5
	Over 2.5~Less than 3 Million Won	99	36.5
	Over 3 Million Won	34	12.5
	Non-response	13	4.8

Shiftwork characteristics of nurses

The average unsocial work hours per week were 30.65 ± 5.33. The average number of unhealthy shift patterns was 4.36 ± 2.38. The overall nurse staffing adequacy was 100%, but there were differences in each ward's degree of staffing. Nurses had low levels of control over shiftwork. The mean scores for the necessity of changes to the roster at short notice, swapping shifts with colleagues, and requests to work specific shifts were 2.03 ± 0.89, 1.89 ± 0.75, and 1.97 ± 1.00 (out of a total of 5), respectively (Table 3).

Private lives of nurses

In terms of family status, a little less than half of the shift nurses were living with parents (41.3%). Regarding demands at home, close to half (44.3%) of all shift nurses had housekeeping responsibilities (44.3%). The mean time spent on housework or childrearing on workdays and holidays was 1.64 ± 1.81 and 5.93 ± 3.42 hours, respectively. More than half of working nurses (66.1%) received domestic help. The means for intrapersonal, interpersonal, and structural constraints were 2.73 ± 0.64, 2.75 ± 0.61, and 3.04 ± 0.64 (out of a total of 5), respectively (Table 3).

Work-life conflict of nurses

The mean score for work-life conflict was 2.77 ± 0.68 (out of a total of 5), the mean score for work interference with work was 3.23 ± 0.85, which was higher than the mean score of 2.30 ± 0.81 for life interference with work. Based on these findings, we can conclude that nurses found their work to be more disruptive than their private life (Table 3).

Table 3. Results of shiftwork characteristics, aspects of private life, and work-life conflict

Categories	Sub-categories	Variables	Mean ± SD/N (%)
Shiftwork characteristics	Work hours	Average number of over 40-hour weeks in a month	2.02
		Unsocial working hours per week	30.65
		Overtime work	0.53±0.56
	Shift pattern	Average number of maximum consecutive shifts per month	6.59±2.01
		Average number of unhealthy shift patterns in a month	4.36±2.38
	Nurse staffing adequacy	Nurse staffing adequacy	100%
	Shift control	Control over specific shifts	2.38±0.69
		Control over specific start & finish times	2.15±0.82
	Shift instability	Required to change roster at short notice	2.03±0.89
		Swapping shifts with colleagues	1.89±0.75
		Requests to work specific shifts	1.97±1.00
Aspect of private life	Family status	Living alone	59 (21.8)
		Cohabiting with sponsor, friends, etc.	39 (14.4)
		Living with parents	112 (41.3)
		Living with children	2 (0.7)
		Living with sponsor, and children	46 (17.0)
		Living with parents, sponsor, and children	13 (4.8)
	Demands at home	Dependent children	61 (22.5)
		Caring for someone with a chronic disease/handicap at home	15 (5.5)
		Caring for family/friend outside home	22 (8.1)
		Housekeeping responsibility	120 (44.3)
	Life events during past year	Divorce	2 (0.7)
		Accidents	7 (2.6)
		Severe illness	8 (3.0)
		Death of important person	16 (5.9)
	Time spent on housework/child rearing on workdays		1.64±1.81
	Time spent on housework/child rearing on holidays		5.93±3.42
	Support at home	Domestic help (available or not)	179 (66.1)
	Leisure constraints	Intrapersonal leisure constraint	2.73±0.64
		Interpersonal leisure constraint	2.75±0.61
		Structural leisure constraint	3.04±0.64
Work-life conflict	Time based work-life conflict	Time based work interference with life	3.23±0.85
		Time based life interference with work	2.30±0.81

Factors influencing work-life conflict of nurses

Multiple regression analyses were conducted to identify the factors that independently related to work-life conflict. Table 4 shows the results of the regression analyses, which were conducted with a total of predictor variables: shiftwork characteristics and aspects of private life. Sex, marital status, and work experience were set as control variables. The regression model was found to be significant (F=6.03, *p*=<.001), and the adjusted coefficient of

determination (Adj R^2), which indicates the explanatory power of the model, was .311. Significant factors related to work-life conflict included control over specific start and finish times of work (β=-0.16, *p*=.019), frequency of swapping shifts with colleagues (β=-0.15, *p*=.025), and leisure constraints (β=-0.39, *p*=<.001).

Table 4. Relationship between shiftwork characteristics, aspect of private life, and work-life conflict

Variables	work-life conflict				
	B	SE	β	t	*p*
(Constant)	1.61	0.41		3.82	<.001
Sex					
Female (vs. male)	-0.14	0.12	-0.07	-1.17	.242
Marital status					
Married (vs. or not)	0.43	0.18	0.29	2.46	.015
Work experience	-0.02	0.01	-0.21	-2.82	.005
**Shift work characteristics**					
Work hours					
Number of over 40-hour working weeks in a month	0.04	0.05	0.06	0.89	.376
Unsocial working hours per week	-0.01	0.01	-0.08	-1.00	.319
Overtime	0.13	0.07	0.11	1.93	.054
Shift pattern					
Number of maximum consecutive shifts over 3 months	-0.01	0.02	-0.03	-0.48	.632
Number of unhealthy shift patterns in a month	0.03	0.02	0.09	1.04	.299
Nurse staffing adequacy	-0.24	0.15	-0.09	-1.61	.110
Shiftwork control					
Control over specific shifts	0.09	0.07	0.09	1.32	.188
Control over specific start & finish times	-0.13	0.06	-0.16	-2.36	.019
Shift instability					
Required to change roster at short notice	0.04	0.05	0.05	0.81	.420
Swapping shifts with colleagues	0.14	0.06	0.15	2.26	.025
Requests to work specific shifts	0.01	0.04	0.01	0.15	.883
**Aspect of private life**					
Family status (vs. living alone)					
Cohabiting with sponsor, friends, etc.	-0.13	0.16	-0.07	-0.83	.408
Living with parents	-0.12	0.11	-0.09	-1.12	.265
Living with children	-0.62	0.51	-0.08	-1.21	.229
Living with sponsor and children	0.04	0.22	0.02	0.18	.860
Living with parents, sponsor and children	-0.19	0.25	-0.06	-0.78	.438
Demands at home	0.02	0.05	0.03	0.31	.756
Time spent on housework/child rearing	0.03	0.03	0.10	1.06	.292
Support at home (vs. yes)	0.08	0.09	0.06	0.95	.344
Leisure constraints	0.47	0.07	0.39	6.86	<.001
Adjusted R^2 = .311, F(*p*) = 6.032 (<.001), VIF = 1.143~5.543					

Note: control variable (sex, marital status, work experience)

Discussions

This study reported an average of 2.02 over 40-hour weeks in a month. This means that for about half every month nurses work more than 40 hours per week as part of their schedule. The nurses in our study reported unsocial working hours to cover an average of 30.65 hours per week. In England, unsocial working hours of nurses are divided into several categories, with Saturday nights and early morning hours paid an additional 50% of basic pay, and Sundays or national holidays, earning nurses an additional 100% of basic pay [21]. However, regardless of the nature of unsocial working hours, only night and holiday working allowances are provided in Korea. The average amount of overtime was found to be 0.53 ± 0.56. Lee, Jeong, Ko, and Kim [22] that the average amount of overtime was 1.32 hours. The occurrence of overtime can be seen as a problem associated with nurse staffing. Inappropriate nurse staffing will result in overtime work.

The average number of unhealthy shift patterns was found to be 4.36 per month. It was frequently confirmed that nurses worked more than three-shift rotations for more than five consecutive days, working with less than 24-hours of rest between shifts, working with less than two nurses on night shifts, a backward rotation working system, etc. In October 2019, guidelines issued for night shifts for nursing staff proposed a night shift planning, including guaranteeing rest (48 hours and over) between consecutive two-night shifts, and limiting periods of consecutive night shifts to less than three days [23]. However, current shiftwork schedules were found to exceed these limits. Therefore, more detailed guidelines for shiftwork, particularly for night shifts, will have to be worked out.

Overall nurse staffing adequacy was found to be 100%. However, nurse staffing adequacy at the ward level showed significant variations from 33% to 162%. The differences among the wards were not only a matter of nurse staffing adequacy, but also contributed to other

problems related to inappropriate nursing services, patient safety, the burden of workload, and turnover. Thus, it can be seen that the overall improvement in this area is necessary.

The scores for control over specific shifts and control over specific start and finish times were 2.38 and 2.15, respectively. Price [24] reported that 55% of respondents showed a low level of satisfaction when asked about the degree of control over the work environment. If welfare is strengthened with measures such as extra graded pay for shifts, providing at least 48 hours of rest between night shifts, and increasing the number of working nurses on night shifts, nurses will have the ability to choose among patterns of shifts that fit their situations. Before implementing the various shift types, the working environment should be restructured to give nurses more control. We found that the scores for the necessity for changes in the roster at short notice, that swapping of shifts with colleagues and requesting to work specific shifts were 2.03, 1.89, and 1.97, respectively. We were able to identify a high frequency of changes to the roster at short notice. Changing shifts with short notice could increase the burden of work on nurses, which could have negative effects on work-life balance. Unstable shifts mean that nurses do not possess enough resources to cope with unexpected work vacancies.

A prior study found that work interference with life was greater than life interference with work [25, 26]. Studies have also found that nurses who work longer hours are more likely to experience interference in life due to work [27]. That is, the more time spent on their work, the greater the experience of work interference in life. A previous study observed that working nights or on weekends interferes with family time, and it is one of the primary reasons for turnover [28]. Unsocial working hours, such as nights or weekend shifts, may interfere with family time, which may increase the degree of work interference with life. As nurses are overloaded with structural constraints, such as shiftwork, it is important to identify and improve factors that can reduce their work-life conflicts. Since, factors such as age and

work experience cannot be altered, it is necessary to look for ways to reduce work-life conflict to improve the nurses’ work environment and prevent turnover in clinical settings.

The lower degree of control over specific start and finish times of shifts makes for a higher degree of work-life conflict. Previous studies have also confirmed the findings of the current study in this regard [29]. It has been shown that when workers are allowed to control their working hours, the work environment and work-life balance improve [30]. While the direct comparison is difficult, this is similar to the fact that non-flexible working hours were shown to increase work-life conflicts [31] and that nurses who have flexible working hours find a better balance between work and life than those with non-flexible working hours [8]. Therefore, diverse shift patterns should be introduced into clinical nursing sites. The higher the frequency of shifts swapped with colleagues, the higher the degree of work-life conflict. Although no prior study was available for a comparison to regard to this finding, it can be surmised that the high frequency of shift changes with colleagues can be a burden to nurses, which is of a similar nature to the higher workload causing higher levels of work-life conflict [9].

This study reported that leisure constraints had an impact on work-life conflict. Previously, Park, Shin, and Shin [32] found that leisure balance was found to have a negative effect on work-life conflict, supporting the current findings. In particular, nurses in their late twenties with less than five years of work experience value leisure and work-life balance. However, in the actual working environment, shifts and weekend work greatly restrict leisure activities. The above-identified factors affecting work-life conflict should be appropriately incorporated into specific plans and policies for improving nursing environments in relation to shiftwork schedules.

Conclusions

The critical results obtained in this study are as follows:

First, the lower degree of control over specific start and finish times of shifts makes for a higher degree of work-life conflict.

Second, leisure constraints had an impact on work-life conflict.

The implications of this study are as follows. First, prior studies that investigated the shiftwork characteristics of nurses mainly measured the subjective variables through nurses' perception. However, in this study, the number of over 40-hour working weeks, unsocial working hours per week, the average number of unhealthy shift patterns, and nurse staffing adequacy were measured as objective variables by receiving the three months shiftwork schedules. It has minimized the recognition bias of nurses and complemented memory inaccuracies. Second, a total of 8 unhealthy shift patterns were derived to measure the number of unhealthy shift patterns by analyzing the shiftwork schedules.

References

1. Albertsen, K., Rafnsdóttir, G., Grimsmo, A., Tómasson, K., & Kauppinen, K. Workhours and worklife balance. Scandinavian J Work Envir & Health, 2008;34(5):14–21.
2. Pichler, F. Determinants of Work-life Balance: Shortcomings in the Contemporary Measurement of WLB in Large-scale Surveys. Social Indicators Res, 2009;92(3):449-69.
3. Pryce, J., Albertsen, K., & Nielsen, K. Evaluation of an open- rota system in a Danish psychiatric hospital: A mechanism for improving job satisfaction and work–life balance. J Nurs Manag, 2006;14(4):282–8. https://doi:10.1111/j.1365-2934.2006.00617.x
4. Lee, T. H., Kang, K. H., Ko, Y. K., Cho, S. H., & Kim, E. Y. Issue and challenges of nurses' workforce policy: A critical review and implication. Korean Academy of Nursing Administration, 2014;20(1):106–16.
5. Korean Ministry of Education. The opportunity to become a nurse expands [Internet]. Sejong-si, Republic of Korea. Korean Ministry of Education; 2018 [cited 25 Nov 2019]. Available from: https://www.moe.go.kr/boardCnts/view.do?boardID=294&boardSeq=75430&lev=0&m=0204
6. Lee, G. J., & Hwang, S. W. Analysis of reemployment status of nurses participated in reemployment support program. J Korean Cont, 2015;15(5):386–402.
7. Trinkoff, A., Geiger-Brown, J., Brady, B., Lipscomb, J., & Muntaner, C. How long and how much are nurses now working?: Too long, too much, and without enough rest between shifts, a study finds. American J Nurs, 2006;106(4):60–71.
8. Jung, M. S., Kim, H. L., & Lee, Y. J. Factors influencing work-life balance in Korean registered nurses. Korean J Occup Health Nurs, 2017;26(2):114–23.
9. Yildirim, D., & Aycan, Z. Nurses' work demands and work-family conflict: A questionnaire

survey. Inter J Nurs Stud, 2008;45(9):1366–78. https://doi:10.1016/j.ijnurstu.2007.10.010

10. Barton., J., Spelten., E., Totterdell., P., Smith., L., Folkard., S., & Costa., G. The Standard Shiftwork Index: A battery of questionnaires for assessing shiftwork-related problems. Work & Stress, 1995;9(1):4–30. https://doi:10.1080/02678379508251582

11. Korean Ministry of Employment and Labor. Health management for shift workers [Internet]. Sejong-si, Republic of Korea. Korean Ministry of Employment and Labor. 2004 [cited 18 Nov 2019]. Available from: http://www.moel.go.kr/local/seoulnambu/info/dataroom/view.do?bbs_seq=1164865560888

12. Korean Confederation of Trade Unions. Opinion of the Korean Confederation of Trade Union on recommendation for work guidelines for healthy shift workers [Internet]. Seoul, Republic of Korea. Korean Confederation of Trade Unions. 2002. [cited 11 Dec 2019]. Available from: http://nodong.org/index.php?filter=search&mid=data_paper&search_keyword=%EA%B5%90%EB%8C%80%EA%B7%BC%EB%AC%B4&search_target=title_content&document_srl=88492

13. Korean National Health Insurance. Guidelines for integrated nursing care services [Internet]. Wonju-si, Republic of Korea. Korean National Health Insurance. 2019. [cited 11 Dec 2019]. Available from: https://www.gov.kr/portal/locgovNews/1917350

14. Shim, M. Y. System of nurses in intensive care unit. [Internet]. 2018. [cited 19 Nov 2019]. Available from: http://www.bosa.co.kr/news/articleView.html?idxno=2083717

15. Jansen, W. H. N., Kant, S. I., Kristensen, J. N. T., & Nijhuis, J. N. F. d: A prospective cohort study. Journal of Occup Enviro Med, 2003;45(5): 479–91. https://doi:10.1097/01.jom.0000063626.37065.e8

16. Takeuchi, T., & Yamazaki, Y. Relationship between work–family conflict and a sense of coherence among Japanese registered nurses. Japan J Nurs Sci, 2010;7(2):158–68.

https://doi:10.1111/j.1742-7924.2010.00154.x

17. Raymore, L., Godbey, G., Crawford, D., & Von Eye, A. Nature and process of leisure constraints: An empirical test. Leisure Sci, 1993;15(2):99–113. https://doi:10.1080/01490409309513191

18. Hubbard, J., & Mannell, R. C. Testing competing models of the leisure constraint negotiation process in a corporate employee recreation setting. Leisure Sci, 2001;23(3):145–63. https://doi:10.1080/014904001316896846

19. Hwang, S. H., & Seo, H. J. The relationship among leisure constraints, leisure constraints negotiation, serious leisure. Korean J Sport Sci, 2009;20(2):298–307.

20. Carlson, D. S., Kacmar, K. M., & Williams, L. J. Construction and Initial Validation of a Multidimensional Measure of Work–Family Conflict. J Vocational Behavior, 2000;56(2):249–76. https://doi:10.1006/jvbe.1999.1713

21. UNISON. Unsocial hours arrangements in the public and private sectors [Internet]. UNISON; 2016 [cited 11 Dec 2019]. Available from: https://www.unison.org/uk/content/uploads/2016/10/Unsocial-hours-benchmarks.pdf

22. Lee, J. S., Jeong, H. J., Ko, H. M., & Kim, H. J. A study on the status of health and medical workers in 2017 to prepare labor conditions and demands for labor cooperation among members of the health and medical industry union. 2017. Korean National Health and Medical Industry Union.

23. Korean Ministry of Health and Welfare. Guidelines for nursing staff's night shifts [Internet]. Korean Ministry of Health and Welfare; 2019 [cited 25 Nov 2019]. Available from: http://www.mohw.go.kr/react/al/sal0101vw.jsp?PAR_MENU_ID=04&MENU_ID=040102&CONT_SEQ=350963&page=1

24. Price, M. Job satisfaction of registered nurses working in an acute hospital. British J Nurs,

2002;11(4):275. https://doi:10.12968/bjon.2002.11.4.10080

25. Ng, L. P., Chen, I. C., Ng, H. F., Lin, B. Y., & Kuar, L. S. Influence of job demands and job control on work-life balance among Taiwanese nurses. J Nurs Manag, 2017;25(6):438–48. https://doi:10.1111/jonm.12482

26. Unruh, L. Y., Raffenaud, A., & Fottler, M. Work-family conflict among newly licensed registered nurses. J Healthcare Manag, 2016;61(2):129–45.

27. Grzywacz, J. G., Frone, M. R., Brewer, C. S., & Kovner, C. T. Quantifying work–family conflict among registered nurses. Res Nurs & Health, 2006;29(5):414–26. https://doi:10.1002/nur.20133

28. Miracle, V., & Miracle, J. The nursing shortage: Why we stay, why we leave, and why we never considered nursing as a career in the first place. Dimensions Crit Care Nurs, 2004;23(5): 236. https://doi:10.1097/00003465-200409000-00014

29. Beutell, N. J. Work schedule, work schedule control and satisfaction in relation to work-family conflict, work-family synergy, and domain satisfaction. Career Develop Int, 2010;15(5):501–18. https://doi:10.1108/13620431011075358

30. Albrecht, S. C., Kecklund, L. G., Rajaleid, K., & Leineweber, C. The longitudinal relationship between control over working hours and depressive symptoms: Results from SLOSH, a population-based cohort study. J Affect Disorders, 2017;215:143-51.

31. Byron, K. A meta-analytic review of work–family conflict and its antecedents. J Vocational Behavior, 2005;67(2):169–98. https://doi:10.1016/j.jvb.2004.08.009

32. Park, J. Y., Shin, J. H., & Shin, K. R. The mediating effects of work-family conflict between leisure balance and marital satisfaction in working women. Korean J Leisure Rec, 2015;39(1):85–97.

Figure Click here to access/download;Figure;Figure (15 June).docx

Nurse	TUR	FRI	SAT	SUN	MON	TUE	WED	TUR	FRI	SAT	SUN	MON	TUE	WED	Total workdays	Number of nurses assigned (A)	Required number of nurse (B)	Nurse staffing adequacy (A)/(B)
	1	2	3	4	5	6	7	8	9	10	11	12	13	14				
Nurse 1	D	D	D	OFF	D	D	D	D	E	OFF	OFF	D	D	D	11	78	11*12=132	0.59
Nurse 2	OFF	E	E	E	N	N	OFF	D	D	D	E	E	E	OFF	11	75	11*12=132	0.57
Nurse 3	N	OFF	D	D	E	E	E	OFF	E	N	N	N	OFF	E	11	75	11*12=132	0.57
Nurse 4	E	N	N	N	OFF	D	D	E	OFF	D	D	E	N	N	12	82	12*12=144	0.57
Nurse 5	D	D	D	OFF	E	E	N	N	N	OFF	OFF	D	D	D	11	78	11*12=132	0.59
Nurse 6	D	OFF	OFF	E	OFF	D	D	OFF	D	D	D	D	OFF	D	9	61	9*12=108	0.56
Nurse 7	OFF	D	E	N	N	N	OFF	D	D	E	OFF	N	N	N	11	76	11*12=132	0.58
Nurse 8	E	E	OFF	D	D	OFF	N	N	N	OFF	E	E	E	E	11	76	11*12=132	0.58
Nurse 9	N	N	N	OFF	D	E	E	E	OFF	N	N	OFF	D	OFF	10	68	10*12=120	0.57
# of day shifts' nurses	3	3	3	2	3	3	3	3	3	3	2	3	3	3				
# of evening shifts' nurses	2	2	2	2	2	2	2	2	2	1	2	3	2	2				
# of night shifts' nurses	2	2	2	2	2	2	2	2	2	2	2	2	2	2				
Total assigned nurses	7	7	7	6	7	7	7	7	7	6	6	8	7	7				

Figure 1. Example of nurse staffing adequacy

Click here to access/download
Supporting Information
questionnaires.docx
