# Supplementary material for: Effects of nurses’ shiftwork characteristics and aspects of private life on work-life conflict
Source: PLoS One. 2020 Dec 1;15(12):e0242379. doi: 10.1371/journal.pone.0242379 (PMC7707512; doi:10.1371/journal.pone.0242379)
Supplement: Attachment — Submitted filename: comments to the author.docx [file pone.0242379.s004.docx]

**Comments to the author**

Indeed, this study is a worth study in measuring the nurses’ shift work characteristics and aspects of private life on work-life conflict.

I would like to suggest the following recommendations to improve the scientific rigor of the study.

1. Mention the appropriate sample size calculation formula used to calculate sample size.
2. In the study it is mentioned that samples were selected based on their willingness other than that have you used any inclusion and exclusion criteria for sample selection.
3. Mention the sampling method.
4. The authors made a good attempt to describe the variables, instruments and their validity.
5. The discussion part appears to be confused, please try to organise the discussion part same as that of the results, by including more studies.
6. Mention the strength and weakness of your study.
7. Try to include the limitations.
8. Write the conclusion properly.
9. Do a language editing.
